# Comparison of Gated and Ungated Black-Blood Fast Spin-echo MRI of Carotid Vessel Wall at 3T

**DOI:** 10.2463/mrms.mp.2014-0133

**Published:** 2015-11-06

**Authors:** Chengcheng ZHU, Martin J GRAVES, Umar SADAT, Victoria E YOUNG, Jonathan H GILLARD, Andrew J PATTERSON

**Affiliations:** 1 University Department of Radiology, University of Cambridge Cambridge, CB2 0QQ, UK; 2 Cambridge Vascular Unit, Cambridge University Hospitals NHS Foundation Trust

**Keywords:** carotid plaque, atherosclerosis, black-blood magnetic resonance imaging, cardiac gating, repeatability

## Abstract

**Purpose:**

Multi-slice ungated double inversion recovery has been proposed as an alternative time-efficient and effective sequence for black-blood carotid imaging. The purpose of this study is to evaluate the comparative repeatability of this multi-contrast sequence with respect to a single slice double inversion recovery prepared gated sequence.

**Materials and Methods:**

Ten healthy volunteers and three patients with Doppler ultrasound defined carotid artery stenosis >30% were recruited. T_1_-weighted (T_1_W) and T_2_W fast spin-echo (FSE) images were acquired centered at the carotid bifurcation with and without cardiac gating. Repeat imaging was performed without patient repositioning to determine the variations in vessel wall measurement and signal intensity due to gating, while negating variations as a result of slice misalignment and anatomical displacement relative to the receiver coil. The distributions and the repeatability of lumen area, vessel wall area, signal and contrast-to-noise ratio (SNR/CNR) of the vessel wall and adjacent muscle were reported.

**Results:**

The T_1_W ungated sequence generally had comparable wall SNR/CNR with respect to the gated sequence, however the muscle SNR was lower (*P* = 0.013). The T_2_W ungated multi-slice sequence had lower SNR/CNR than the gated single slice sequence (*P* < 0.001), but with equivalent effective wall CNR (*P* = 0.735). Vessel area measurements using the gated/ungated sequences were equivalent. Ungated sequences had better repeatability in SNR/CNR than the gated sequences with borderline and statistically significant differences. The repeatability of T_2_W wall area measurement was better using the ungated sequences (*P* = 0.02), and the repeatability of the remaining vessel area measurements were equivalent.

**Conclusions:**

Ungated sequences can achieve comparable SNR/CNR and equivalent carotid vessel area measurements than gated sequences with improved repeatability of SNR/CNR. Ungated sequences are good alternatives of gated sequences for vessel area measurement and plaque composition quantification.

## Introduction

Carotid atheroma is an established risk factor for subsequent stroke and transient ischemic attack. High resolution magnetic resonance imaging (MRI) of the carotids can visualize high-risk morphologic factors such as lipid core and hemorrhage and improve patient risk stratification.^1^ Multi-contrast protocols including black-blood T_1_-weighted (T_1_W) and T_2_W fast spin-echo (FSE) and bright blood time-of-flight (TOF) angiography are established for plaque measurement and composition characterization^2^ and have been validated against histology.^3,4^

Carotid studies have extensively used sequences which were analogous to cardiac imaging, whereby blood suppression was performed using gated triggering and double inversion recovery preparation.^5,6^ These sequences allowed single slice acquisition per pass,^7^ and lead to significant scan times which disparaged their clinical adoption.^8^ The in-plane pulsatile motion of the carotid vessel wall during a cardiac cycle is of the order of 0.4 mm which is close to the in-plane resolution of current MRI techniques^9^ and subsequent studies have shown that ungated sequences produced comparable signal-to-noise ratio (SNR) and plaque measurements while the scan time was reduced.^10^ Further optimization was done by Yarnykh and Yuan, who described how the double inversion recovery preparation could be adapted for multi-slice acquisition when combined with ungated FSE sequences.^11^ Ungated sequences have subsequently gained prominence and have been successfully applied in a number of clinical studies.^8,12,13^

Ungated sequences allow fixed repetition times (TRs) to generate consistent T_1_ recovery. A practical constraint limiting the clinical adoption of gated sequences was the high occurrence of bradycardia and arrhythmia in symptomatic patient cohorts. This leads to variable image contrast in gated images, which can affect plaque characterization.

The reproducibility of gated sequences has been well investigated^14–17^; reports on the reproducibility of ungated sequences are rare.^18,19^ To date, no study has systematically compared the signal intensity and vessel morphology variations due to gated and ungated triggering.

In this study, we aim to examine the repeatability of carotid vessel wall measurements and signal intensity using multi-contrast MRI with and without cardiac gating.

## Materials and Methods

Ten healthy volunteers (5 males, age 23–40 years) and three patients with known carotid atheroma (2 males, age: 70–85 years, minimum stenosis of 30–49%) identified by Doppler ultrasound were recruited. The study was conducted following local ethical committee approval, and all participants gave informed written consent.

### MRI acquisition

Imaging was performed on a 3T whole body MRI system (Signa HDx, GE Healthcare, Waukesha, WI, USA) using a 4-channel phased-array neck receiver coil (PACC, Machnet BV, Elde, The Netherlands). To capture the comparative variations in image contrast and morphology attributed to the gated/ungated sequences, the subject was rescanned without repositioning, thereby minimizing variations as a result of slice misregistration and anatomical displacement relative to the surface coil. Movement artifact was minimized using a dedicated vacuum-based head restraint system (VAC-LOK Cushion, Oncology Systems Limited, Shropshire, UK).

Two-dimensional (2D) TOF MR angiography was used to localize the position of the carotid bifurcation. Multi-contrast protocols which comprised fat suppressed T_1_W and T_2_W FSE, with and without cardiac gating were prescribed. The acquisition parameters are listed in Table 1. The ungated multi-slice double inversion recovery (DIR) sequence was implemented as described by Yarnykh and Yuan,^11^ and comparisons were performed against a standard single-slice, gated, double inversion recovery prepared sequence. One slice acquisition was prescribed per TR for both gated and ungated T_1_W acquisitions, and one slice and four slices were acquired, respectively, per TR period for the gated and ungated sequences for the T_2_W acquisition. For gated sequences, a patient/volunteer specific delay time was prescribed to make the acquisition in diastolic phase. A spatially matched noise-only sequence was prescribed, this sequence consisted of a modified gradient echo sequence where the amplitude of the radiofrequency pulse was set to zero. The receiver gain was fixed between these scans and the slice position/thickness was identical. Scan parameters of the noise-only image are listed in Table 1. This noise quantification method is advantageous over traditional methods which measure the noise in a background artifact-free region, because subtle motion artifacts are commonly present in carotid scans.^20,21^

### Image analysis

To maintain independence assumptions required for subsequent statistical analysis, a single vessel segment was analyzed. In total, 13 vessel locations were included. Lumen and outer wall boundary were segmented by a reviewer using the Vessel Mass software (Leiden University Medical Centre, Leiden, The Netherlands). Lumen area (LA) and wall area (WA) were automatically calculated from the segmented contours.

Regions of interest (ROIs) were manually drawn on an adjacent region of sternocleidomastoid muscle, and noise was quantified using a spatially matched region on the noise-only image. The SNR of muscle, wall, and lumen were computed as SI/σ × 0.695, where SI was the signal intensity of the muscle, wall and lumen, and σ was the standard deviation of noise. The multiplier 0.695 corresponds to a four-channel coil correction.^22^ Wall contrast-to-noise ratio (CNR) was defined as wall SNR-lumen SNR. Effective CNR was defined as (CNR)/(T_slice_)^1/2^ as adopted from previous published work,^23^ where T_slice_ was the scan time (in minute) per slice.

### Statistical analysis

Formal assessment using the Shapiro-Wilk’s test found that the distributions were not Gaussian-like, i.e., normally distributed, consequently the Wilcoxon signed-rank test was used to assess differences in distributions. Repeat 1 was chosen to compare relative SNR, CNR, and vessel wall measurements between the gated and ungated sequences. The distribution central tendency and spread were summarized using the median and interquartile range.

The repeatability of the signal intensity metrics and vessel area measurements were summarized using Bland Altman summary statistics.^24^ Systematic bias between repeat scans was defined as the mean of the pair-wise differences. Repeatability was summarized as the 95% limits of agreement (LOA) range, defined as 2 × 1.96σ, where σ is the standard deviation of the pair-wise differences.

Permutation tests were designed to test the null hypothesis H_0_ that the ungated sequence had less variation between repeat scans than the gated sequence. The pairwise difference vector between repeat 1 and 2 was computed for each signal intensity and vessel area metric. The test was performed to compare the observed ratio of variation in the difference vectors *r* = *var_diff−gated_*/*var_diff−gated_*. The observed ratio was first computed (*r_obs_*) and this was compared to the distribution of random permutations of the difference vectors where random sampling was performed without replacement. Statistical inferences were computed using a non-exact implementation (performing 50000 unique permutations).^25^

Statistical significance was defined as a *P* value <0.05. All the statistical analyses were performed using the statistical programing language R version 2.7.0 (The R Foundation of Statistical Computing, Vienna, Austria).

## Results

**Fig. 1.**
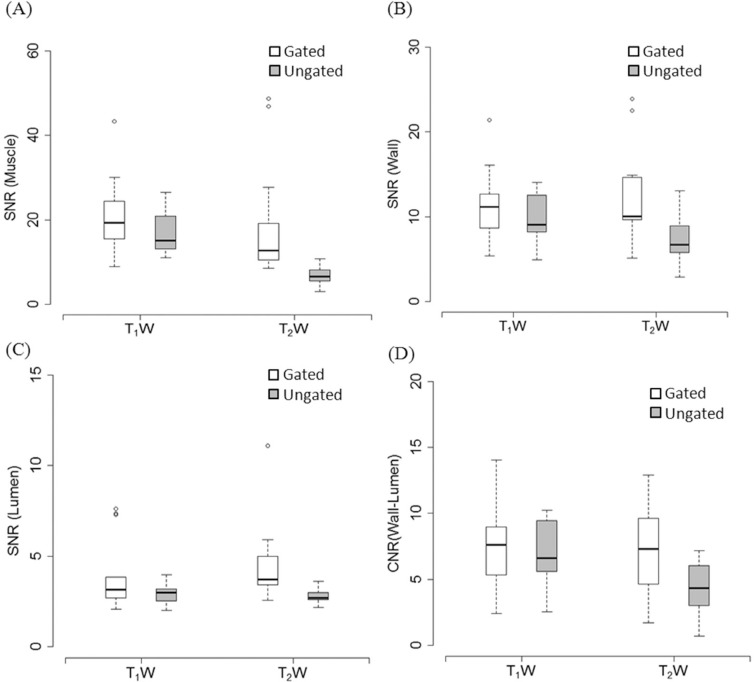
Signal-to-noise ratio (SNR) and contrast-to-noise ratio (CNR) comparison between gated and ungated sequences in T_1_-weighted (T_1_W) and T_2_W images. Blank boxes show the gated sequences, and gray boxes show the ungated sequences.

Comparisons of relative SNR and CNR in vessel wall, lumen, and adjacent sternocleidomastoid muscle are shown in Fig. 1 and summarized in Table 2. A comparison of the single slice T_1_W acquisition suggests the SNR and CNR were comparable with the exception of the observed difference in SNR of muscle (*P* = 0.013). T_1_W vessel area measurements were equivalent. The T_2_W comparisons demonstrated that the multi-slice ungated sequence had consistently lower SNR and CNR (*P* < 0.001); however, when correcting for the effective acquisition time the CNR was equivalent (*P* = 0.735). The vessel area measurements were also equivalent.

A comparison of the relative repeatability of the techniques is summarized in Table 3. A qualitative comparison of SNR and CNR suggests the repeatability (as defined by the 95% LOA range) is better using the ungated sequences, whereas the vessel area measurements were equivalent. Borderline significant improvements in repeatability were observed in SNR and CNR using the ungated methods, with T_1_W wall SNR and T_2_W muscle SNR showing statistical significant improvements (*P* = 0.049 and *P* < 0.001, respectively). When comparing the T_2_W sequences, the ungated images demonstrated a smaller LOA range which was partially due to the overall smaller SNR/CNR. However, note that the normalized percentage of LOA range (LOA range/median × 100%) was also smaller (muscle SNR: 7.6% vs. 52.3%; wall SNR: 22.4% vs. 33.3%; wall CNR: 34.9% vs. 42.5%). The vessel area measurements were equivalent, for the exception of the T_2_W wall area which also demonstrated a significant improvement in repeatability (*P* = 0.02).

**Fig. 2.**
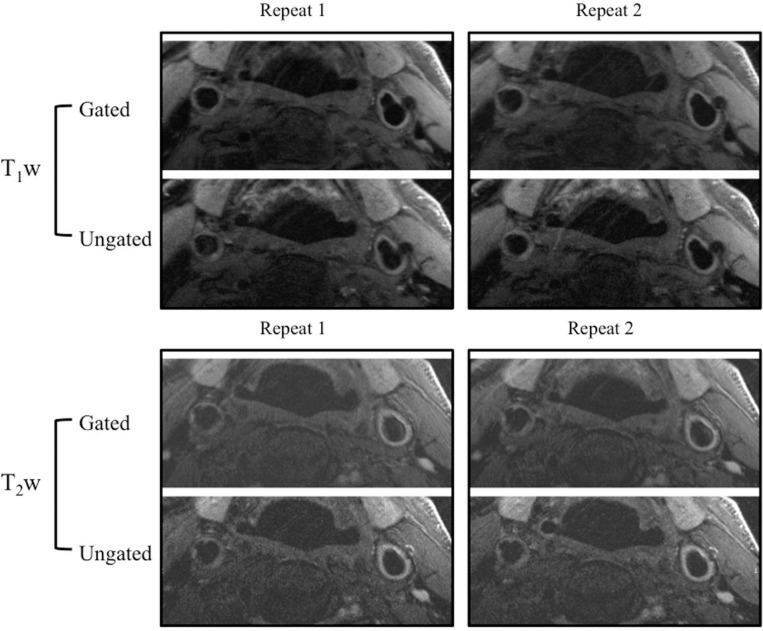
T_1_-weighted (T_1_W) and T_2_W images with and without gating in a patient.

Representative images acquired using the gated and ungated sequences are shown in Fig. 2.

## Discussion

Although multi-slice DIR prepared ungated black-blood FSE sequences have been successfully used for carotid vessel wall imaging,^8,12,13^ the influence of ungated triggering on the repeatability of image contrast and vessel area measurements have not been well investigated. To our best knowledge, this is the first study to systematically compare the repeatability of SNR/CNR and vessel area measurements between gated and ungated sequences in carotid vessel wall imaging.

We found ungated sequences had comparable SNR/CNR to the gated sequence in T_1_W single slice acquisition. In T_2_W images, ungated sequences had lower SNR/CNR than the gated sequence due to the multi-slice acquisition (4 slices per TR vs. 1 slice per TR). The reduction of SNR in multi-slice acquisition is well known to be attributed to slice cross-talk.^11^ However, no significant difference was found between the effective wall CNR using gated and ungated sequences in T_2_W images. Vessel area measurements were equivalent using gated/ungated sequences in both T_1_W and T_2_W images. These findings agreed with Mani et al.,^10^ who previously found ungated sequences produced comparable SNR and plaque measurements in T_2_W multi-slice protocols.

In addition, we found the ungated sequences had generally better repeatability in SNR/CNR than the gated sequences with borderline and statistically significant differences. The 95% LOA range of the ungated sequence was much smaller (<60%) than the gated sequences. The borderline *P* value might attribute to the relatively small sample size (n = 13) in this study. Such bigger signal intensity variations of gated sequences were possibly because the variable heart rate of the subjects leads to variable T_1_ recovery time between each TR. Most of the subjects included were healthy volunteers (10/13), who had normal cardiac function with relatively stable heart rate. We did not record the heart rates of the study subjects, which is a limitation of this study. The repeatability of SNR/CNR could be poorer in symptomatic patients who often had bradycardia and arrhythmia using gated sequences.

To achieve an accurate and a reliable plaque component classification and quantification, continuous efforts have been devoted to developing automated segmentation tools.^26,27^ Such techniques based on statistical pattern recognition algorithms ideally require repeatable contrast mechanisms. The results from our study suggest ungated sequences have substantially less signal intensity variation in repeat scans, thus providing more consistent image contrast for reliable plaque composition characterization. Future studies on the repeatability of automatic segmentation methods using gated/ungated sequences are needed to validate such findings.

Clinical evaluation of carotid artery disease requires robust and repeatable definition of vessel area measurements for arterial stenosis and plaque burden quantification. It was comforting to note vessel area measurements obtained from the ungated sequences were comparable or better than the gated sequences. This seems somewhat counterintuitive, as the gated sequences were defined to acquire data during a diastolic phase, whereas the gated sequences sampled randomly throughout the cardiac phases. One possible explanation could be the pulsatile motion was comparable to the spatial resolution.^9^ In this study we used an in-plane resolution of 0.39 mm × 0.39 mm, which was comparable with previous studies (range 0.4 mm to 0.6 mm).^3,4,10^ Although it is possible to increase the resolution (for example, to 0.2 mm) to study the potential improvement of gated acquisition, such high resolution images need long scan time and lead to significantly lower SNR, which is impractical in a clinical setting.

Permutation tests were performed to compare the repeatability of both approaches as the benefits of these approaches are that the inherently account for the non-parametric nature of the observed data, the statistical inferences are entirely data driven and are not dependent on standard assumptions, and these techniques are more sensitive to standard F-tests, which are known to require large sample sizes to observe significant differences in variation.

## Conclusion

Ungated sequences can achieve comparable SNR/CNR and equivalent carotid vessel area measurements to the gated sequences with improved repeatability of SNR/CNR. Ungated sequences are good alternatives to gated sequences for vessel area measurement and plaque composition quantification.

## Figures and Tables

**Table 1.** Acquisition parameters

	T_1_W ungated/gated	T_2_W ungated/gated	Noise image (GRE)
TR (ms)	800/1 R-R	2500/2 R-R	20
TE (ms)	13.3	50	5.7
Slice per pass	1/1	4/1	N/A
FOV (cm)	10 × 10	10 × 10	10 × 10
Matrix	256 × 256	256 × 256	256 × 256
Slice thickness (mm)	2	2	2
NEX	3	3	3
Fat suppression	Yes	Yes	N/A
ETL	10	12	N/A
Scan time (s)*	61.44/76.8	40/128	15.36

* The gated acquisition assumes a heart rate of 60 bpm and scan times are quoted per slice. T_1_W, T_1_-weighted; GRE, gradient echo; N/A, not available; FOV, field of view; NEX, number of excitations (averages); ETL, echo train length

**Table 2.** Distributions of T_1_-weighted (T_1_W) and T_2_W signal-to-noise ratio (SNR) and contrast-to-noise ratio (CNR) and prescribed lumen and wall area measurements comparing gated and ungated sequences

	T_1_-weighted median [IQR]		*P*	T_2_-weighted median [IQR]		*P*
	
	Gated	Ungated		Gated	Ungated	
Muscle SNR	19.3 [8.9]	15.2 [7.8]	0.013	12.8 [8.7]	6.6 [2.6]	<0.001
Wall SNR	11.2 [4.0]	9.0 [4.3]	0.340	10.0 [4.9]	6.7 [3.1]	<0.001
Lumen SNR	3.2 [1.1]	3.0 [0.7]	0.09	3.7 [1.6]	2.7 [0.4]	<0.001
Wall CNR	7.6 [3.6]	6.6 [3.8]	0.685	7.3 [4.9]	4.3 [3.0]	<0.001
Wall CNReff*	6.6 [3.2]	6.4 [3.7]	0.893	4.9 [3.3]	5.2 [3.6]	0.735
Lumen area (cm^2^)	0.446 [0.145]	0.452 [0.112]	0.127	0.410 [0.141]	0.385 [0.295]	0.735
Wall area (cm^2^)	0.277 [0.279]	0.293 [0.132]	0.635	0.272 [0.283]	0.337 [0.129]	0.224

* Note that the wall CNReff was calculated by normalizing the distribution by the square root of the acquisition time ratio. IQR, interquartile range

**Table 3.** Repeatability of gated and ungated sequences in signal-to-noise ratio (SNR)/contrast-to-noise ratio (CNR) and lumen/wall area measurements. Cells show the bias and 95% limit of agreement (LOA) range

	Gated		Ungated		*P* value
	
	Bias	95% LOA range	Bias	95% LOA range	
T_1_-weighted					
Muscle SNR	0.11	11.5	0.58	3.5	0.069
Wall SNR	0.06	5.5	0.29	2.08	0.049
Wall CNR	−0.10	4.5	0.15	2.4	0.066
Lumen area	0.03	0.13	−0.01	0.07	0.198
Wall area	−0.01	0.11	0.01	0.10	0.399
T_2_-weighted					
Muscle SNR	0.11	6.7	−0.03	0.5	<0.001
Wall SNR	−0.04	3.0	0.11	1.5	0.055
Wall CNR	0.18	3.1	0.11	1.5	0.081
Lumen area	0.02	0.11	0.02	0.08	0.365
Wall area	0.01	0.15	−0.01	0.08	0.020
